# The Beginnings of the London Hospital

**Published:** 1914-09-26

**Authors:** 


### THE BEGINNINGS OF THE LONDON HOSPITAL.

### A New Light on its Early Days.

Mr. Morris commenced his "History of the London Hospital" with a meeting of the promoters in the bar-parlour of The Feathers Tavern, Cheapside, on September 23, 1740, but remarks that there had evidently been previous meetings, for Mr. Harrison then "delivered in the lease of the house, which had been already taken for the intended infirmary." This was the house in Featherstone Street, which was rented at £16 per annum, with liberty to quit at six months' notice, and which was opened to the public November 3, 1740. But it was soon found inadequate to the requirements of the charity, and after six months (in May 1741) a move was made to Prescott Street, Goodman's Fields, that being near the usual abodes of manufacturers and seafaring men in the merchant service, and far from any other hospital.

By the discovery of a printed "Proposal for raising an Infirmary by voluntary subscription and contribution for the more immediate relieving the sick and diseased poor," evidently published prior to September 1740, new light is thrown upon the early history of the movement which resulted in the establishment of the London Infirmary, as it was then called. The Proposal proceeds: "We have more hospitals, and those better regulated, than any other country. Some have been nobly endowed and graciously given by Kings, others of more modern institution erected and supported by voluntary subscription, as St. George's Hospital and the Infirmary (at Westminster). But all these are inadequate to the numbers seeking assistance, and attempts have been made to provide for those unable to obtain admission for lack of beds. Though charity money has been frequently misapplied, it is proposed to set on foot a subscription list to raise 200 guineas at least, which it is conceived will answer the expense of necessaries for about 1,000 paupers per annum in their several courses of cure (without nourishment or lodging). Wherefore this Charity will be conducted upon the system of out-patients in other hospitals. But if 300 guineas are raised, the Infirmary will be able to take in, lodge, and diet at the rate of 120 patients per annum, who most need it. It will be necessary for a convenient House to be taken, which for manifold reasons should be in or near Moorfields,

| | |
|---|---|
| Not more in rent, taxes, and contingent charges than ... ... ... ... ... ... ... | £25 p.a. |
| A servant man and a motherly woman to reside constantly in it, whose wages, board, etc., together should not exceed ... ... ... | £48 p.a. |
| For fire, candles, soap, etc. ... ... ... | £16 |
| For furnishing 3 rooms— 1 for the managers to meet in. 1 for the physician. 1 for the surgeon to examine and dress the patients in ... ... ... ... | £30 |
| For medicines for the aforesaid paupers ... | £80 |
| | £199 |

It is proposed that—

Each subscriber of 5 guineas shall have a vote in the management of this Infirmary.

When 200 or even 150 guineas have been subscribed the subscribers shall elect a Treasurer.

A President, Vice-President, and Committee of Managers shall be chosen, and a Committee then appointed to meet as thought best for the management of the said intended Infirmary.

One Physician shall be elected to attend gratis every morning from 8 to 10, who shall be of character, ability, and experience. One Surgeon shall be appointed in like manner: none to be elected who has not served his whole apprenticeship in one of the hospitals or infirmaries of this City. A Book shall be kept containing each patient's name, business, abode, case, issue, etc. No money or present shall be taken from patients or their friends. No person shall be admitted without a printed order from a manager. No soldiers, the servants of gentlemen who are not subscribers, nor persons affected with the venereal disease, shall be admitted to partake of this charity.

The Infirmary shall give advice to all poor people: if their cure depends only on bleeding, cutting issues, or such easy means, the surgeon shall perform such operations without entering their names, provided they come in the prescribed hours. These general heads, if found deficient, can be altered or added to by subscribers at their meeting. It shows that no money can be perverted from the intended uses, so that gentlemen may more readily contribute to it."

A subscription form accompanied the Proposal.

It is evident that the hundred guineas which had been subscribed by September 23, 1740, only sufficed for the establishment of a dispensary, and it is certain that no patients were received into the infirmary during the first year of its existence, for no beds or bedding had been provided, and no nurse had been engaged in that period. Just a year after its inauguration a salaried resident apothecary was appointed, previous to which date the only residents were the "servant man and motherly woman" (not matron), to look after the house.

Since no paid apothecary was provided for in the Proposal, and no salary for that officer is entered in the items of the first year's expenditure, it is probable that Mr. Josiah Cole, one of the original promoters, gave his services, like the physician and surgeon. Noteworthy, too, is the rule that no surgeon was to be elected except he had served his whole apprenticeship (seven years) in one of the hospitals or infirmaries of the City. The proviso was peculiar to the London Infirmary, was probably intended to exclude candidates from Scotland, where the period of apprenticeship was of only five years' duration, and supports the statement of Highmore that the surgical character had always been its predominant feature.

But of still greater interest is the light thrown by the Proposal upon the then estimate of institutional expenditure, and the comparison of the relative cost of in- and out-patients. Two hundred guineas were expected to meet the necessary expenses of providing for 1,000 out-patients, the cost per head for medicines working out at 1s. 7¼d., and establishment charges at 2s. 4½d., making a total cost of 3s. 11¾d. for each out-patient.

On the other hand, three hundred guineas were estimated sufficient to lodge, diet and provide medicine for 120 in-patients (nothing appears to have been allowed for out-patients in this contingency) at a cost of £2 12s. 6d. for each in-patient. Mr. Morris states that at the end of the first year 127 in-patients had been treated; but the items of the first year's expenditure fail to support the statement, the amounts expended upon furniture, provisions, candles, and soap being reconcilable only with the treatment being confined to out-patients. The promoters estimated the cost of the latter for the first year at £199; their actual expenditure was £206 5s. 6d.

